# Deep ultraviolet fluorescence microscopy of three-dimensional structures in the mouse brain

**DOI:** 10.1038/s41598-023-35650-2

**Published:** 2023-05-26

**Authors:** Deepa Kamath Kasaragod, Hidenori Aizawa

**Affiliations:** Department of Neurobiology, Graduate School of Biomedical and Health Sciences, Hiroshima University, 1-2-3 Kasumi, Minami-ku, Hiroshima, 734-8553 Japan

**Keywords:** Fluorescence imaging, Neuroscience

## Abstract

Three-dimensional (3D) imaging at cellular resolution improves our understanding of the brain architecture and is crucial for structural and functional integration as well as for the understanding of normal and pathological conditions in the brain. We developed a wide-field fluorescent microscope for 3D imaging of the brain structures using deep ultraviolet (DUV) light. This microscope allowed fluorescence imaging with optical sectioning due to the large absorption at the surface of the tissue and hence low tissue penetration of DUV light. Multiple channels of fluorophore signals were detected using single or a combination of dyes emitting fluorescence in the visible range of spectrum upon DUV excitation. Combination of this DUV microscope with microcontroller-based motorized stage enabled wide-field imaging of a coronal section of the cerebral hemisphere in mouse for deciphering cytoarchitecture of each substructure in detail. We extended this by integrating vibrating microtome which allowed serial block-face imaging of the brain structure such as the habenula in mouse. Acquired images were with resolution high enough for quantification of the cell numbers and density in the mouse habenula. Upon block-face imaging of the tissues covering entire extent of the cerebral hemisphere of the mouse brain, acquired data were registered and segmented for quantification of cell number in each brain regions. Results in the current analysis indicated that this novel microscope could be a convenient tool for large-scale 3D analysis of the brain in mice.

## Introduction

Light microscopy has been an indispensable tool for the structural study of the brain. Three-dimensional (3D) cellular scale imaging techniques that improve our understanding of the brain architecture are crucial to elucidate the structural and functional changes occurring in the normal and the pathological conditions. Recently there has been an surge of interest in 3D imaging of the brain as a bridge to link the microscopic and macroscopic information of the neural circuits^1,2^.

Thick tissue sample scatters light efficiently, and so traditionally 3D reconstruction of the tissue involved 2D sectioning, followed by imaging of the stained sections for better contrast. This procedure was labour-intensive and also involved error-prone 3D reconstruction due to lack of accurate registration information and other tissue preparation artefacts^3^. With the advances in the whole brain staining and microscopy techniques, 3D imaging has adopted a combination of section and serial imaging of the block surface for sectioning tomography^4^. For example, fluorescent microscopy schemes like micro-optical sectioning tomography (MOST) adopts serial block-face imaging using resin embedded samples by thinning slicing sections using a diamond knife^4^. Serial block-face microscopy for 3D imaging uses automated high-resolution microscopy using confocal, two-photon or structured illumination microscopy by combining optical and mechanical sectioning in a serial fashion^1,5,6^. However, those technologies are complex and require a great deal of optical expertise and expense to set up and utilize for 3D imaging, which do not make them a convenient choice, for large-scale deployment in majority of the laboratories. Since the reference map onto which the gene expression and neural circuit can be mapped became available for the model animals such as mouse^7–9^, cost-effective solution for sectioning tomography is demanding for prevalent application of 3D imaging in the neuroscience.

A new method using deep ultraviolet light known as microscope using ultraviolet surface excitation (MUSE) was recently developed especially for 2D imaging of tissue samples^10–12^. This fluorescence microscope makes use of two aspects of DUV light. Firstly, when excited with DUV light, large Stokes shift in the visible spectrum is seen with most of the conventional fluorophores and secondly, strong absorbance of the DUV light and hence low penetration in the tissue samples is achieved. Surface excitation of fluorescence using DUV light provides the optical sectioning ability to be used for imaging thick block of samples using a simple epifluorescence configuration in the optical setup.

In this paper, we aimed to obtain non-contiguous planar information with significant interval along z-axis to obtain interspersed but three-dimensional distribution of the cells, represented as cell number and density in the specific subregions in the tissue block. This is especially effective in analysing the nervous tissue in which the functionally heterogeneous subregions are distributed in space. We present a simple optical setup for block-face imaging combined with DUV illumination. This method was compatible with 3D imaging of the mouse brain towards quantitative analysis at cellular scale resolution. It is expected that this simplified microscope would be established as a useful tool for 2D/3D microscopy in neuroscientific applications.

## Materials and methods

### Spectrophotometry of the fluorescent probes

To assess the spectral compatibility with DUV fluorescence microscope, the absorption and the excitation spectra of the fluorescent probes Hoechst 33258 (0.08 mg/ml, H341, Dojindo Laboratories, Kumamoto, Japan), propidium iodide (0.1 mg/ml, P1304MP, Invitrogen, USA) and Alexa Fluor 594-conjugated antibody (1:50, ab150076, Donkey anti-rabbit IgG H&L, Abcam plc., Cambridge, UK) were obtained using spectrophotometer microplate reader (Varioskan LUX, ThermoFisher Scientific, Waltham, MA, USA). The absorption spectrum (240–800 nm range) and emission spectrum (400–800 nm range) with excitation at 280 nm were measured using UV light transparent 96-well plate (UV-Star, Greiner Bio-One, Longwood, FL, USA).

### Animals

All procedures and experiments were performed in accordance with ARRIVE guidelines (https://arriveguidelines.org). The study was approved by the committees of Hiroshima University for the animal experiments (approval number A22-139) and gene recombination experiments (approval number 2022-90). All methods were carried out in accordance with relevant guidelines and regulations. Four male C57BL6/J (Japan SLC, Inc., Hamamatsu, Japan) and two male heterozygous Emx1-Cre mice^13^ were used for 2D and 3D imaging, respectively. Three to nine-month -old mice were group-housed under specific pathogen-free conditions with free access to food and water. A 12:12 h light–dark cycle of illumination was maintained under the regulation of the temperature and humidity in the range of 18–25 °C and 40–60%, respectively. They were anesthetized by ketamine/xylazine (100 mg/kg, *i.p.*) and transcardially perfused with 4% paraformaldehyde in 0.1 M phosphate buffered saline (PBS). The brains were post-fixed in the same fixative at 4 °C overnight.

### Tissue staining

For the staining of the 2D sections, we incubated the sections in the dye solutions followed by wash in PBS three times. Reagents and concentrations used in the study was as follows: propidium iodide (5 µg/ml, P1304MP, Invitrogen, USA). For whole brain staining with Hoechst 33258, the osmotic shock method was used as described previously^1,14^. Briefly, fixed brains were immersed in staining solution (PBS containing 10 mg/ml Hoechst 33258, 0.1% TritonX-100, and w/v sucrose: Day1—10%; Day2—20%; Day3 to Day7—30%; and Day8—0%) at 55 °C with gentle shaking. For staining of the block-surface of the mouse brain in situ, the bath of the vibrating microtome was filled with propidium iodide solution (5 µg/ml in PBS)^5^. Propidium iodide stained cytoarchitecture visualized both the nucleus and the Nissl bodies^5,15^.

For immunohistochemistry, 50 μm-thick coronal sections of the mouse were washed in PBS three times for 10 min each at room temperature (RT) on a shaker. Following the 0.5% Triton X-100 in PBS for 2 h at RT, sections were incubated with anti-tyrosine hydroxylase (TH) antibody (1:250, rabbit polyclonal, AB152, Sigma Aldrich) overnight at 4 °C. Signals were visualized with secondary antibody (donkey anti-rabbit IgG H&L; Alexa Fluor 594; ab150076, Abcam plc., Cambridge, UK). Some sections were counterstained with Hoechst 33258. Sections were wet-mounted on glass slide with quartz coverslip (Labo-USQ, 20 mm × 20 mm × 0.5 mm thickness, Daiko Seisakusho, Osaka, Japan).

### Wide-field DUV microscope setup

**Figure 1:**
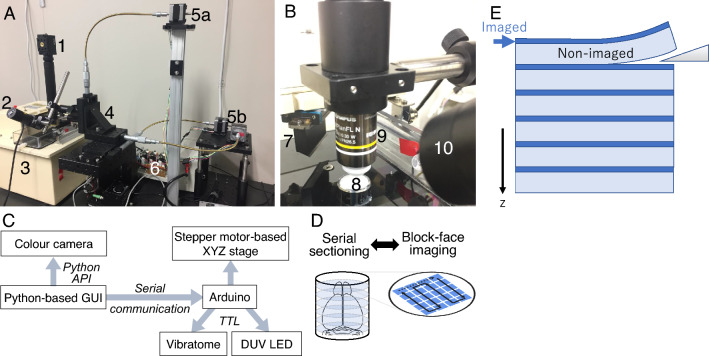
Overview of the 3D-DUV microscope. (**A**) A photo of the entire microscope system showing a combination of the optical parts (1, 2 for camera and light source, respectively), vibrating microtome (3) and motorized stage (4–6 for 3-axes translating stage, stepper motors and their driver unit, respectively). (**B**) A photo of the magnified view of the imaging part showing orientation of the cutting blade (7) and sample stage (8) of the vibrating microtome with optical parts including objective lens (9) and light source (10). (**C**) A schematic of the imaging and serial sectioning parts integrated via microcontroller (Arduino). (**D**) A schematic of the workflow design of serial sectioning and block-face imaging. (**E**) A schematic of the tissue sample embedded in the agarose to show that only the surface of the sample block (dark blue) but not the deeper part (light blue) is imaged. Upon sectioning by the thickness larger than the previously imaged depth, new surface will be imaged consecutively.

Optical design of the DUV fluorescence microscope is similar to that of conventional epifluorescence microscope, but with fewer optical components (Fig. 1A). Side illumination with oblique incidence of light onto the specimen was used (Fig. 1B, 60° angled relative to the vertical plane of the microscope). A multi-spectral detection scheme with spectrally separated fluorophores in RGB channels was adopted using a colour camera (GS3-U3-123S6C-C, FLIR Integrated Imaging Solutions, Inc., OR, USA). The optical sectioning ability of the DUV fluorescence microscope is due to the strong absorption of the DUV light by the molecules and thus the limited depth of penetration of light^10^. Our DUV microscope design further exploited the oblique light illumination for excitation with water immersion objective for increased angle of refraction and lowered depth of penetration of DUV light^11^.

Light emitting diode (LED) at wavelength 280 nm (incident power on the sample as approximately 8.13 mW, Quark Technology, Okayama, Japan) was used as light source. UV grade fused silica biconvex lens (f = 35 mm) was used to converge the DUV light and limit the area of the illumination. The imaging was carried out using the 10X objective lens (UMPLFLN 10XW, NA = 0.3, Olympus Corporation, Tokyo, Japan). The field of view with the 10X objective was approximately 1.3 mm × 1.0 mm. Image acquisition was performed at default camera settings for exposure time of 30 ms.

For wide-field imaging, a custom-designed motorized stage controlled by Arduino Mega 2560 microcontroller (Arduino LLC, Somerville, MA, USA) was used as a modified version of the open-source based stage controller developed previously^16^ (Fig. 1C). The XY stage translates the microscope head to obtain multiple images as mosaics to cover the entire mouse brain section in the chosen anatomical plane. A flexible shaft was connected to 500 µm/revolution micrometer actuated linear translator (X and Z axes: 25 mm translation stage PT1/M; Y axis: 50 mm translation stage XR50P/M, Thorlabs, Japan). Fifty mm translation stage was used for the Y-axis to allow for the complete evacuation of the imaging head for tissue slicing after each block-face imaging.

Custom-fabricated couplers were used to connect the shaft to the stepper motors (PK243M-01B; Oriental Motors, Japan) and the micrometers of the translation stage. For the stepper motor step size of 0.9°, a minimum resolution of 1.25 µm was obtained with the translators. The master control was carried out using the custom-written software using Python3 which communicates with the Arduino through serial communication (Fig. 1C). The number of X and Y translations required to cover the entire tissue section or tissue section of interest was calculated based on the information provided in mouse brain reference atlas in stereotaxic coordinates^17^ with a 20% overlap between the tiles. The parameters corresponding to each of the imaging session was described using YAML files^18,19^.

The widefield imaging capability was extended to carry out whole brain imaging through automated serial block-face imaging (Fig. 1D). This was done by integrating the microscope with a vibrating microtome (Compresstome VF-700-OZ-120, Precisionary Instruments, Greenville, NC, USA) which performs serial sectioning followed by block-face imaging (Fig. 1B). Microtome was controlled using a relay switch (Y14H-1C-5DS; Hsin Da Precision Co. Ltd, Taiwan) through Arduino Mega 2560 to automatically start and stop the sectioning of the tissue after completion of the wide-field data of the previous tissue section (Fig. 1C). Since the acquired images reflect the signal originating from the limited depth (dark blue in Fig. 1E) which is much smaller than the sectioning thickness by vibrating microtome (light blue in Fig. 1E), we obtained non-contiguous planar information with significant interval along z-axis.

### Imaging with laser scanning confocal and epifluorescent microscopes

We used laser scanning confocal microscope (FLUOVIEW FV1200, Olympus Corporation) exciting Hoechst 33258 with 405 nm laser and propidium iodide with 559 nm laser (10X water immersion objective lens with NA = 0.3, pinhole size of 1 Airy Unit). Imaging with epifluorescent light were done either a using custom built microscope of the configuration similar to DUV microscope but with the optical filters in place (Emission filter—FF01-446/523/600/677–25, Semrock; Excitation filter—FF01-390/482/563/640–25, Semrock) and 405 nm LED light source (LED4D254, Thorlabs) or macro zoom fluorescence microscope (MVX10, Olympus Corporation).

### Axial profiling of the microscope setup using fluorescent microbead

Four µm microbeads (TetraSpeck, Invitrogen, USA) were used for profiling the DUV microscopy setup with 10X water immersion objective (NA = 0.3, Olympus, Japan). The XYZ profiles were obtained as an average of images for 10 microbeads embedded in 2% agarose. Gaussian fit and full width at half maximum were obtained using custom python scripts.

### Serial block-face imaging with DUV

3D imaging was carried out with the sample placed in the sample holder of the vibrating microtome. The tissue sample was glued onto the sample holder and was then embedded in 2.0% agarose (low EEO Agarose, A0576, Sigma-Aldrich) inside the sample holder. To allow faster setting of the agarose, the bath surrounding the sample holder was filled with chilled PBS solution kept at 4 °C. Then, serial slicing was carried out to expose the tissue surface for imaging. For staining of the brain samples in the microtome bath, PBS is replaced with the respective dye solution as the immersion medium of the objective in the bath. For staining of the block-surface in situ, propidium iodide solution (5 µg/ml) was filled in the bath of the vibrating microtome.

Upon exposing the block-face surface of the tissue section of interest to start the image acquisition, images per field of view (FOV) were acquired as per the averaging and Z-stack values input for all the FOVs across the entire wide-field imaging. The XYZ stages for positioning the imaging unit were brought back to the original location after completion of an entire wide-field image acquisition of the exposed tissue section.

To allow the microtome blade to slice off the next section, the imaging head was translated (Y axis) 50 mm away from the original position. The microtome then sectioned the exposed block-face at the desired thickness of 50 µm (imaging of the entire mouse habenula) or 100 µm (imaging of the whole mouse brain) followed by returning of the blade back to the original position. The imaging head was then moved back to the original position and the whole process was repeated till the imaging of the entire block of tissue sample was carried out over serial sections of desired thickness.

### Quantification of the optical sectioning thickness

Nissl-stained fluorescence signals using propidium iodide was used for isolating each cells using the co-registered DUV image and confocal z-stacks. Z-stacks were obtained using confocal microscope at depth separation of 2 µm using 10X and 20X water immersion objective lenses (UMPLFLN 10XW, NA = 0.3 and UMPLFLN 20XW, NA = 0.5, Olympus Corporation, Tokyo, Japan). Same specimen was imaged using DUV microscope and co-registered images were obtained. 1345 cells were segmented using a custom developed ImageJ macro. Confocal z-stacks were used to locate the depth of the individual cells and the optical sectioning thickness was assessed by sorting the cells in DUV images based on the depth information. DUV signal attenuation with depth was modelled based on the Beer’s law and the 1/e depth of the exponential fit was used to obtain the estimate of the optical sectioning thickness^11^. Statistical measure by averaging over a large number of cells was used for this analysis as varying signal intensities across and within cells for Nissl bodies were observed. Image similarity of the DUV image with the different images of the confocal z-stack at various depth was obtained using Pearson correlation coefficient to corroborate the estimated value.

### Data analysis

The DUV microscopy images were denoted as RGB for colour images comprising of red, green and blue channels, respectively. Unless otherwise specified, signals in red channel were shown in grey-scale intensity levels. The image processing steps included colour correction and flat-field correction. Extended depth of focus imaging was optionally carried out if multiple z-stack images were acquired for each FOV. Wide-field mosaic was obtained using Microsoft Image Composite Editor for each of the brain sections. ImageJ was used for further data analysis as described below (https://imagej.nih.gov/ij/)^20^.

For 3D quantitative analysis of the cell numbers in the mouse habenula, cells labelled with propidium iodide were counted using Cell Counter plugin of ImageJ for 10 serial sections taken with uniform spacing of 150 µm across the serial sections acquired for unilateral habenula with referring to the anatomical landmarks such as the third ventricle and stria medullaris.

For prototypical whole brain analysis, we consecutively imaged 117 block-face images with interval of 100 µm, spanning from the olfactory bulb to the cerebellum. Eight-bit grayscale images obtained were then converted to 16-bit images for automatic cell counting. Semi-automatic alignment of images to Allen brain reference template [Allen Mouse Common Coordinate Framework (CCF v3)]^21^ followed by segmentation and automated cell counting (AP =  − 0.92 mm from Bregma) were done using WholeBrain pipeline software^22,23^. The percentage of cell counts was calculated for each brain region and represented as sunburst graph.

### Statistical analyses

Statistical analyses were performed using the statistical package R (R Development Core Team 2011), jamovi 1.2 (The jamovi project 2020, retrieved from https://www.jamovi.org). Binary comparisons for two groups were assessed using two-tailed paired t-tests, and comparisons between three or more groups were performed using Welch's one-way ANOVA followed by Tukey’s honestly significant difference (HSD) test for multiple comparisons. Distribution of the signal intensity at different anterio-posterior level of the mouse brain was examined by Shapiro–Wilk test of normality. Statistical significance was defined as *p* < 0.05.

## Results

**Figure 2:**
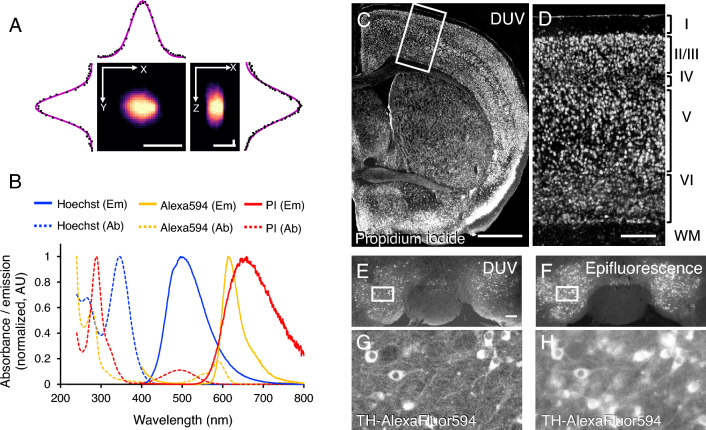
DUV imaging of the mouse brain stained with synthetic dye and immunohistochemistry. (**A**) Reconstructed DUV images of a fluorescent bead (TetraSpeck, 4 µm in diameter) projected onto the X–Y and X–Z spaces with intensity plots (raw and Gaussian-fitted data as shown by black dots and magenta solid lines, respectively). (**B**) Line plots of the normalized absorbance (Ab, dashed) and emission (Em, solid) spectra of the synthetic dyes (Hoechst 33258 and propidium iodide in blue and red, respectively) and Alexa Fluor 594-conjugated anti-rabbit IgG antibody (orange). (**C**, **D**) DUV images of a coronal section of the mouse brain stained with propidium iodide showing Nissl-like staining of the cell bodies of neurons in the cerebral hemisphere and in the cerebral cortex. Panel (**D**) is a magnified view of a boxed area in (**C**). (**E**, **F**) Tiled images of the mouse midbrain imaged with DUV (**E**, **G**) and epifluorescence (**F**, **H**) showing expression of tyrosine hydroxylase (TH) visualized with Alexa Fluor 594. Panels (**G**) and (**H**) are magnified views of the boxed areas in panels (**E**) and (**F**), respectively. Scale bars represent 4 µm (**A**), 1 mm (**C**), 200 µm (**D**), 200 µm (**E**, applies to **F**). Roman numerals on the right of panel D indicate the layers of the cerebral cortex. *WM* White matter.

We first examined the axial resolution of the DUV microscope using 3D profiling of the fluorescent microbeads. Full widths at half maximum for X, Y, and Z axes (4.19, 4.24, and 32.5 µm, respectively) were determined from the virtual slices along XY and YZ planes (Fig. 2A). This analysis confirmed the 3D resolution high enough for imaging single cells.

Next, we examined compatibility of the synthetic dye and fluorescent probe used for immunohistochemistry with DUV. Spectrophotometry revealed that synthetic dyes frequently used in Hoechst 33258 (nucleus) and propidium iodide for Nissl staining (nucleus and Nissl substance in the perikaryal area) in neuroscience^20^ had absorption peaks in DUV range (peaked at 266 and 289 nm for Hoechst 33258 and propidium iodide, respectively) with emitting fluorescence in green and red spectrum (peaked at 496 and 659 nm as shown by blue and red in Fig. 2B, respectively). We also examined donkey anti-rabbit IgG conjugated with Alexa Fluor 594 conventionally used in immunohistochemistry and found its absorption and fluorescence peaks at 278 nm and 613 nm, respectively (orange in Fig. 2B). These results suggested that fluorescent probes conventionally used in neuroscience could be, at least in part, applicable to DUV imaging.

We applied those probes to the mouse brain section to image the wide field of neural structure covering a hemisphere at cellular resolution by DUV microscope. Propidium iodide excited by DUV emitted signal localized in the cell bodies predominantly in grey matter over white matter on a coronal section of the mouse brain (Fig. 2C). Images exhibited Nissl-like pattern with intense signal in the perikaryal region of the neuronal cell bodies showing six-layered cytoarchitecture in the cerebral cortex (Fig. 2D). Furthermore, this wide field imaging with DUV revealed distribution of the neurons with cell-type specific marker such as tyrosine hydroxylase (TH), a protein marker for dopaminergic neurons in the midbrain. Indeed, immunohistochemistry using anti-TH antibody visualized with Alexa Fluor 594 showed cell bodies were distributed specifically in the substantia nigra pars compacta and ventral tegmental area in the midbrain (Fig. 2E). These images were obtained with high resolution allowing to identify detailed localization of the immunoreactivity in the perikaryal regions, cytosol and proximal part of neuropils (Fig. 2G). Upon imaging of the same sections with conventional epifluorescence microscope, we found images with less contrast and low signal-to-noise ratio probably due to the emitted light from the optical plane out of focus (Fig. 2F,H). Upon combining this with Hoechst 33258 nuclear staining for multi-colour labelling, we observed specific distribution of the Alexa Fluor 594 and Hoechst 33258 in the cytosol (magenta in Supplementary Fig. 1) and nucleus (cyan in Supplementary Fig. 1), respectively. These results indicated the wide-field DUV microscope which we developed in the current study were useful tool to examined neural structures labelled with fluorescent probes for nuclear and Nissl staining and immunohistochemistry at cellular resolution.

**Figure 3:**
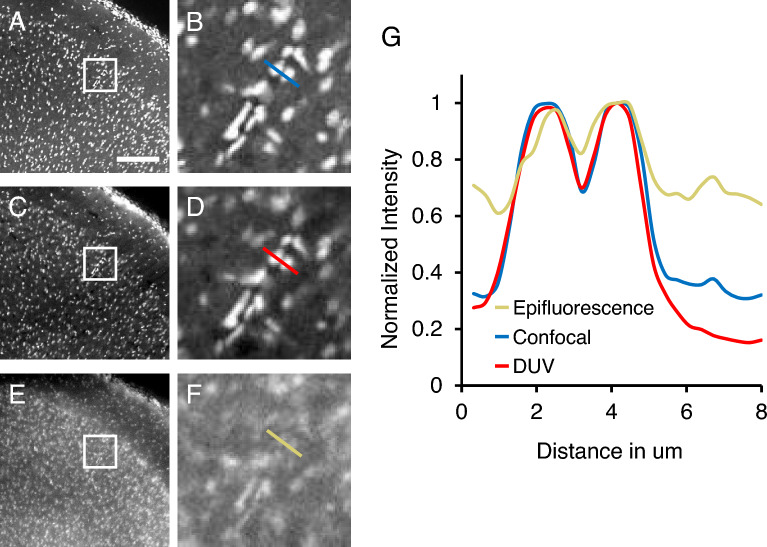
Block-face imaging of a thick block of mouse brain over exposed region stained with Hoechst 33258. (**A**–**F**) Coronal sections of the secondary motor area of the mouse cerebral cortex imaged by the confocal microscope (**A**, **B**), DUV microscope (**C**, **D**), and epifluorescence microscope using visible light (405 nm) for excitation (**E**, **F**). Panels (**B**, **D**) and (**F**) are magnified view of the boxed areas in panels (**A**, **C**, **E**), respectively. (**G**) Normalized intensity plots of the signals acquired along the coloured lines in panel (**B**, **D**, **F**). Scale bar, 100 μm (applies to panels **C** and **E**).

Since thick tissue block is likely to have more significant light scattering than cut section as examined above, we addressed applicability of wide-field DUV microscope to the block-face imaging of the mouse brain tissue (Fig. 3). Vibrating microtome exposed the surface of the secondary motor cortex imaged with laser scanning confocal (Fig. 3A,B), DUV (Fig. 3C,D) and epifluorescent microscope (Fig. 3E,F). This was supported by intensity profiling of signals on these cells showing significant difference between three types of microscopes (Fig. 3G, one way ANOVA, F_(2,40.8)_ = 8.55, *p* < 0.001; Tukey’s HSD post hoc test, *p* < 0.01 for DUV vs epifluoresence).

**Figure 4:**
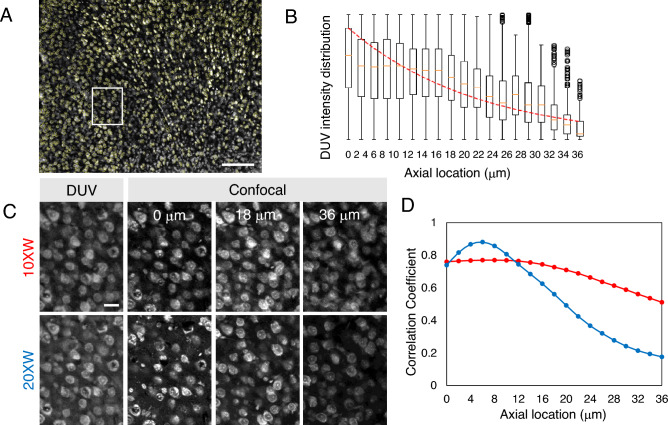
Quantitative assessment of the optical sectioning thickness in the mouse brain. (**A**) A coronal section showing the segmented cells outlined in yellow. (**B**) A box plot of the DUV signal intensities for each depth and the exponential curve fit to obtain the 1/e depth for the estimation of the optical sectioning thickness. (**C**) Magnified view of the boxed area in A imaged using 10 × and 20 × water objective lens. Co-registered DUV and confocal images from the z-stack at depth of 0, 18 and 36 μm are shown. (**D**) A line plot of correlation coefficient between DUV image and confocal z-stack at each axial location using 10 × (red) and 20 × objective lens (blue). Scale bars represent 200 µm (**A**) and 25 µm (**C**).

To quantitatively assess the optical sectioning thickness, we performed a novel experiment using the method which models the DUV intensity attenuation with depth based on Beer’s law and defines the 1/e depth of the intensity attenuation as the estimated optical sectioning thickness as previously described^11^. The results showed that the optical sectioning thickness was estimated to be 20 µm based on the 1/e depth of the DUV intensity of the cells sorted based on depth information from the confocal z-stacks (Fig. 4A,B). Similarity between DUV image and confocal image taken at each depth was assessed with different optical zoom and numerical aperture (Fig. 4C). Quantitative analysis revealed that correlation coefficient between DUV image and a confocal optical section deeper than 20 µm from the surface reduced in line with results of the above analysis (Fig. 4D). This confirms the block-surface optical sectioning capability of the DUV microscope.

**Figure 5:**
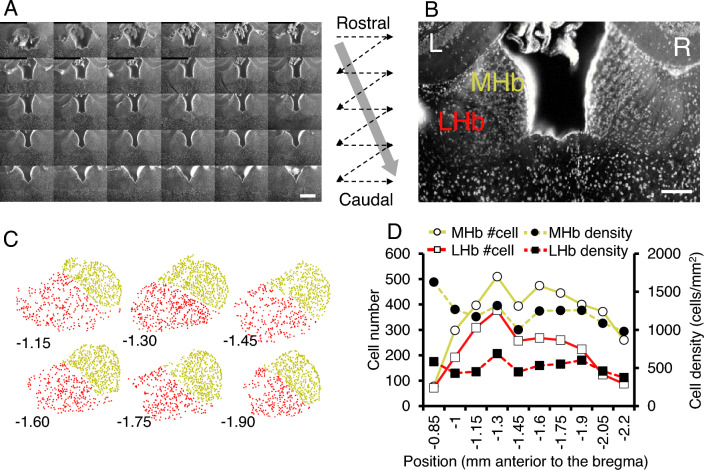
Quantitative analysis of the 3D dataset for the mouse habenula acquired by DUV sectioning tomography. (**A**) Montage of the coronal sections showing 30 DUV images of the entire extent of the mouse habenula stained with propidium iodide (step size, 50 µm). (**B**) A coronal section of the bilateral habenula showing the medial (MHb) and lateral habenula (LHb) as substructures. (**C**) Mapping of the cells onto entire extent of the MHb (yellow) and LHb (red) along rostro-caudal axis as revealed by Cell Counter plugin in ImageJ. (**D**) Line plots of the cell count (solid lines) and density (dashed lines) in MHb (yellow) and LHb (red). Values in abscissa represent the rostro-caudal position of the coronal section relative to the bregma. L, left; R, right. Scale bar, 500 µm (**A**) 200 µm (**B**).

We first addressed applicability of our methods to the prototypical case with limited area and promising signal recognition by manual cell counting and registration to the structure. To obtain 3D information from the specific brain structure, we applied the block-face DUV imaging with serial sectioning to the mouse habenula, a longitudinal structure distributing along the rostro-caudal axis (Fig. 5A). Block-surface imaging with in situ Nissl-like staining with propidium iodide exhibited the medial (MHb, yellow in Fig. 5B) and lateral habenula (LHb, red in Fig. 5B). Acquired images were at cellular resolution high enough to allow quantification of the number of neuronal cell bodies in a series of sections (Fig. 5C). Analysis revealed greater cell number and density in MHb than LHb with statistical contrast (Fig. 5D, two-tailed paired t-test, t test, t_(9)_ = 6.75, *p* < 0.001 for cell number and t_(9)_ = 6.75, *p* < 0.001 for cell density). Although cell quantification and studying the substructure heterogeneity in habenula is a very important topic, there are few previous studies that quantified the density of cells/neurons in the lateral and medial habenula in the mice. Although a previous study reported a significant difference of cell density between medial and lateral habenula in rats, few studies addressed it in mice^24^.

**Figure 6:**
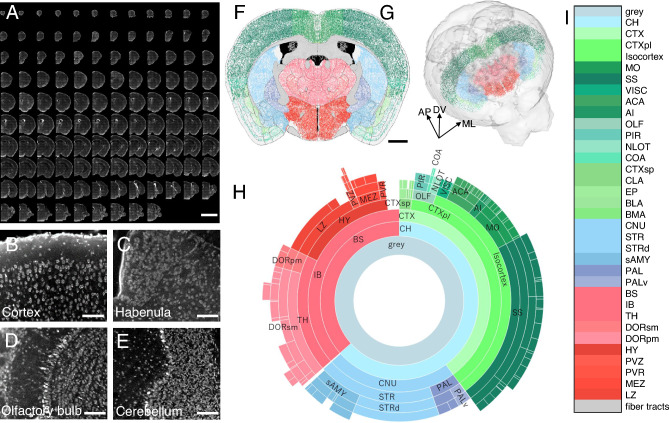
Quantitative analysis of the 3D dataset for the mouse whole brain acquired by DUV sectioning tomography. (**A**) Montage of the coronal sections showing 117 DUV images of the entire extent of the mouse whole brain stained with propidium iodide (step size, 100 µm). (**B**–**E**) DUV images of the mouse cerebral cortex (**B**), habenula (**C**), olfactory bulb (**D**) and cerebellum (**E**) showing layered (**B**, **D**, **E**) and nuclear (**C**) structures. (**F**, **G**) Mapping of the identified neuronal cell bodies mapped onto a coronal plane (**F**) and 3D model of mouse brain atlas (**G**). (**H**, **I**) A sunburst plot (**H**) of the cell count mapped onto the brain structures colour-coded as shown in I. AP, anterior–posterior axis. DV, dorso-ventral axis. ML, medio-lateral axis. Scale bar, 5 mm (**A**) 200 µm (**B**, applies to **C**–**E**), 1.5 mm (**F**). *CH* Cerebrum, *CTX* Cerebral cortex, *CTXpl* Cortical plate, *MO* Somatomotor areas, *SS* Somatosensory areas, *VISC* Visceral area, *ACA* Anterior cingulate area, *AI* Agranular insular area, *OLF* Olfactory areas, *PIR* Piriform area, *NLO* Nucleus of the lateral olfactory tract, *COA* Cortical amygdalar area, *CTXsp* Cortical subplate, *CLA* Claustrum, *EP* Endopiriform nucleus, *BLA* Basolateral amygdalar nucleus, *BMA* Basomedial amygdalar nucleus, *CNU* Cerebral nuclei, *STR* Striatum, *STRd* Striatum dorsal region, *sAMY* Striatum-like amygdalar nuclei, *PAL* Pallidum, *PALv* Pallidum, ventral region, *BS* Brain stem, *IB* Interbrain, *TH* Thalamus, *DORsm* Thalamus, sensory-motor cortex related, *DORpm* Thalamus, polymodal association cortex related, *HY* Hypothalamus, *PVZ* Periventricular zone, *PVR* Periventricular region, *MEZ* Hypothalamic medial zone, *LZ* Hypothalamic lateral zone.

To extend the results in the above prototypical analysis, we set out more comprehensive analysis covering the whole mouse brain followed by cell counting fully automated by WholeBrain pipeline^22^.We acquired the image datasets from the whole mouse brain to analyse the histological data with annotation of detailed brain structures stained with propidium iodide (Fig. 6A). As in the prototypical analysis examining the habenula, high quality data were obtained from the brain structures (Supplementary Fig. 2) ranging from the olfactory bulb (Fig. 6B) through the cerebral cortex (Fig. 6C) and the habenula (Fig. 6D) to the cerebellum (Fig. 6E). Within a single plane in the middle of entire anterio-posterior extent, five different field of views with fixed depth of focus showed signal-to-noise ratio high enough to discern each of the cellular nucleus (Supplementary Fig. 3). The staining of propidium iodide was uniform along the anterior–posterior axis of the mouse brain, since we observed mean signal intensity sampled from six rectangular fields at each level followed Gaussian distribution (Shapiro–Wilk test of normality, W = 0.965, *p* = 0.001) and did not show statistically significant difference (Welch’s one-way ANOVA, F_(22, 41.8)_ = 0.317, *p* = 0.997) (Supplementary Fig. 4). Cell count data was mapped onto coronal planes (Fig. 6F) and 3D annotation space (Fig. 6G) of the Allen brain atlas to segment the anatomical subregions. This allowed the quantification of the number of cells in a hierarchy of brain regions as represented by sunburst plot (Fig. 6H,I).

## Discussion

Microscope in the current study is different from the conventional microscope such as confocal microscope with optical sectioning ability in that it images a surface of the block-face to obtain the structural information of the tissue at a given depth. By consecutive sectioning using vibrating microtome to expose new and deeper block-face for imaging, three-dimensional information of the tissue is thus obtained. Block-face imaging using epifluorescent microscope can result in blurred image due to the emitted light coming from the plane in and out of focus^25^. To address this, we adopted DUV light source which attenuates in the plane deeper than the superficial part of the tissue thus providing optical sectioning capability for block-face imaging.

We combined DUV with vibrating microtome which enables the sectioning of the fixed samples without intensive preparation and embedding of the samples in advance. We aimed to obtain non-contiguous planar information with significant interval along z-axis (e.g., 50 µm) to obtain interspersed but three-dimensional distribution of the cells represented as cell number and density in the specific subregions in the tissue block. This is especially effective in analysing the nervous tissue in which the functionally heterogeneous subregions are distributed in space.

Similar approach was adopted by a previous article in which the DUV was combined with rotary microtome to image the block-face of the samples embedded in paraffin wax following intensive tissue processing such as dehydration, clearing and embedding^26^.Although it allows imaging with finer z-resolution due to the capability to cut thinner sections (by 1 µm) than the current study, we believe that microscope in the current study has advantage in obtaining interspersed information covering major brain regions distributed in the forebrain quickly. Registration of the cellular information to the reference brain atlas using pipeline software further supports its use in processing a large number of samples as frequently found in group comparison with different genotypes and drug treatments in rodent studies.

The underlying principles of DUV microscope are the same as that of the recently developed microscopy with MUSE^10,11^. MUSE has been proposed as a rapid tool for obtaining histology like images for pathology labs. In this study, we show the possible extension of the utility of MUSE/DUV microscope as an alternate for conventional epifluorescence microscope used in neuroscience research for block-face imaging. DUV microscope presented here has a simple optical design that could be convenient alternative to the conventional microscope in research labs for both 2D and 3D microscopy^12^.

From a design perspective, oblique illumination is utilized for DUV microscope unlike the objective lens system for excitation and emission light collection in the conventional fluorescence microscope design. Objective lens with higher numerical aperture has benefits for higher magnification and collection of more light from the samples. But high numerical aperture lens has shorter working distance which can limit the angle of incident light illumination from a design perspective.

Estimated optical sectioning thickness in the current study is slightly higher than the one measured in DUV microscope reported previously^11^ for similar microscope configuration. Since these two studies have difference regarding the samples (fixed mouse brain and unfixed human breast tissue) and staining (exclusive labelling of cell nuclei and labelling of both nuclei and perikaryal Nissl substance), it is likely that differential distribution of the subcellular signal and DUV light scattering in the tissue contributes to the estimated optical sectioning thickness. Further quantitative assessment under different sample conditions would address these points in the future.

It is essential to create the list of the dyes with high fluorescence yield for excitation using DUV spectrum. Previous studies reported that DUV light excited a range of fluorophores including synthetic dyes such as Hoechst 33258, propidium iodide^10^ and quantum dots^27^. In the current study, we showed that immunohistochemistry with Alexa Fluor dye conjugated with antibody was compatible with DUV imaging. In addition to the antibody, Alexa Fluor dyes exhibited high photostability and brightness and were applied to labelling of the anterograde and retrograde tracers widely used in the analysis of neural circuits^28^. Further analysis of the compatibility of those reagents with DUV would enhance the applicability of DUV imaging in neuroscience.

For the application of the block-face imaging to the DUV imaging, whole brain staining with antibody must be essential in advance. Since whole brain staining with antibody were reported with intensive tissue processing for clearing to facilitate antibody penetration, reduced tissue integrity would be a significant concern upon sectioning in block-face imaging^29,30^. Future study is needed to screen the clearing procedure compatible for vibratome sectioning to address this point.

Whether or not UV would pose a problem for tissue imaging might be a concern in general. In the current study, we typically used 6–8 mW of LED power for a brief exposure time of 30 ms, which did not seem to possess problems in tissue imaging for the range of dyes which have explored. Indeed, we observed a preserved cytoarchitecture of the mouse brain with frequent DUV imaging as in the conventional microscopy under visible light. DUV microscopy for fluorescent probes used in immunohistochemistry showed rather better signal-to-noise-ratio as compared to the epifluorescent microscopy. In line with this, a previous study on live cell imaging using DUV light have shown that UV phototoxicity is not a concern for live cell imaging to study phenomena such as mitosis and motility for UV exposure for as long as 45 min^31^.

Illuminating the tissue with DUV also involves the significant presence of autofluorescence coming from the endogenous UV excitable biomolecules attributed to excitation of the aromatic amino acid, nicotinamide adenine dinucleotide hydrogen (NADH) and collagen^32^. Although these molecules are excitable in the range from 220 to 295 nm, their emissions are limited from 280 to 350 nm and outside of the visible spectrum. Thus, it is unlikely that they affect the emitted fluorescence of exogenous fluorophores used in the current study. The fluorescence of the endogenous biomolecules with an improved contrast for brain imaging in cancer imaging was recently shown^33^. In the brain samples, white matter autofluorescence with DUV excitation is significant^34^. Although we did not spectrally separate the autofluorescence in the white matter in the current study, extraction of the signal reflecting the autofluorescence from the specific brain structure such as white matter might be helpful to demarcate the anatomical landmark to gain better insight into the brain structure.

Within the diffraction limited regime, use of the fluorophores with shorter wavelength of the DUV spectrum has the added advantage of better spatial resolution, while capability of microtome in cutting thin section will limit resolution along z-axis in block-face imaging. For example, rotating and freezing microtome commonly used in the histological analysis allows finer sectioning than vibrating microtome, although they require additional processing of the tissues in advance of sectioning such as dehydration, clearing and embedding^26^. In contrast, vibrating microtome used in this study allows sectioning of the fixed tissue without extensive tissue embedding, which is beneficial to preserve more antigenicity in the tissue to make it compatible with immunohistochemistry. Taking the cellular resolution analysis of the mouse brain in the current study into consideration, sectioning tomography with a combination of DUV and vibrating microtome could be a useful solution to analyse the structural and neurochemical properties of the brain.

## Conclusions

We developed a novel microscope for wide-field imaging of 3D structure of the mouse brain using DUV illumination. With integration of the motorised stage, this microscope could cover a coronal section of the entire hemisphere stained with synthetic dyes and immunohistochemistry for protein localization. Imaging of 3D structure in mouse brain was done upon block-face imaging with serial sectioning by vibrating microtome. We showed prototypical analysis of the cell distribution in the entire mouse brain with reference to the standardized mouse brain atlas. Results in the current analysis indicated that this novel microscope could be a convenient tool for large-scale 3D analysis of the mouse brain.

## Supplementary Information

Supplementary Information.

## Data Availability

The data sets used in the current study are available from the corresponding author on reasonable request.
