# Supplementary material for: Deep ultraviolet fluorescence microscopy of three-dimensional structures in the mouse brain
Source: Sci Rep. 2023 May 26;13:8553. doi: 10.1038/s41598-023-35650-2 (PMC10219975; doi:10.1038/s41598-023-35650-2)
Supplement: Supplementary file 1 — Supplementary Information. [file 41598_2023_35650_MOESM1_ESM.pdf]

Supplementary Information

Deep ultraviolet fluorescence microscopy of three-dimensional structures in the mouse brain

Deepa Kamath Kasaragod and Hidenori Aizawa

Department of Neurobiology, Graduate School of Biomedical and Health Sciences, Hiroshima University, Hiroshima, Japan

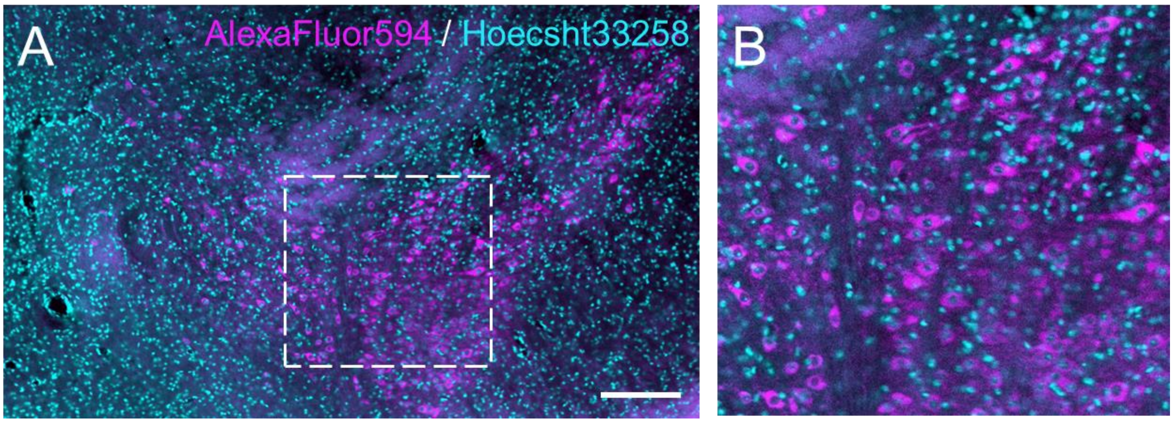

Supplementary Figure 1. A, Multi-colour DUV image of tyrosine hydroxylase visualized by AlexaFluor594-conjugated IgG (magenta) and DNA in the nucleus with Hoechst 33258 (cyan). Scale bar represent 200 μm. Panel B is the magnified view of the boxed area in panel A.

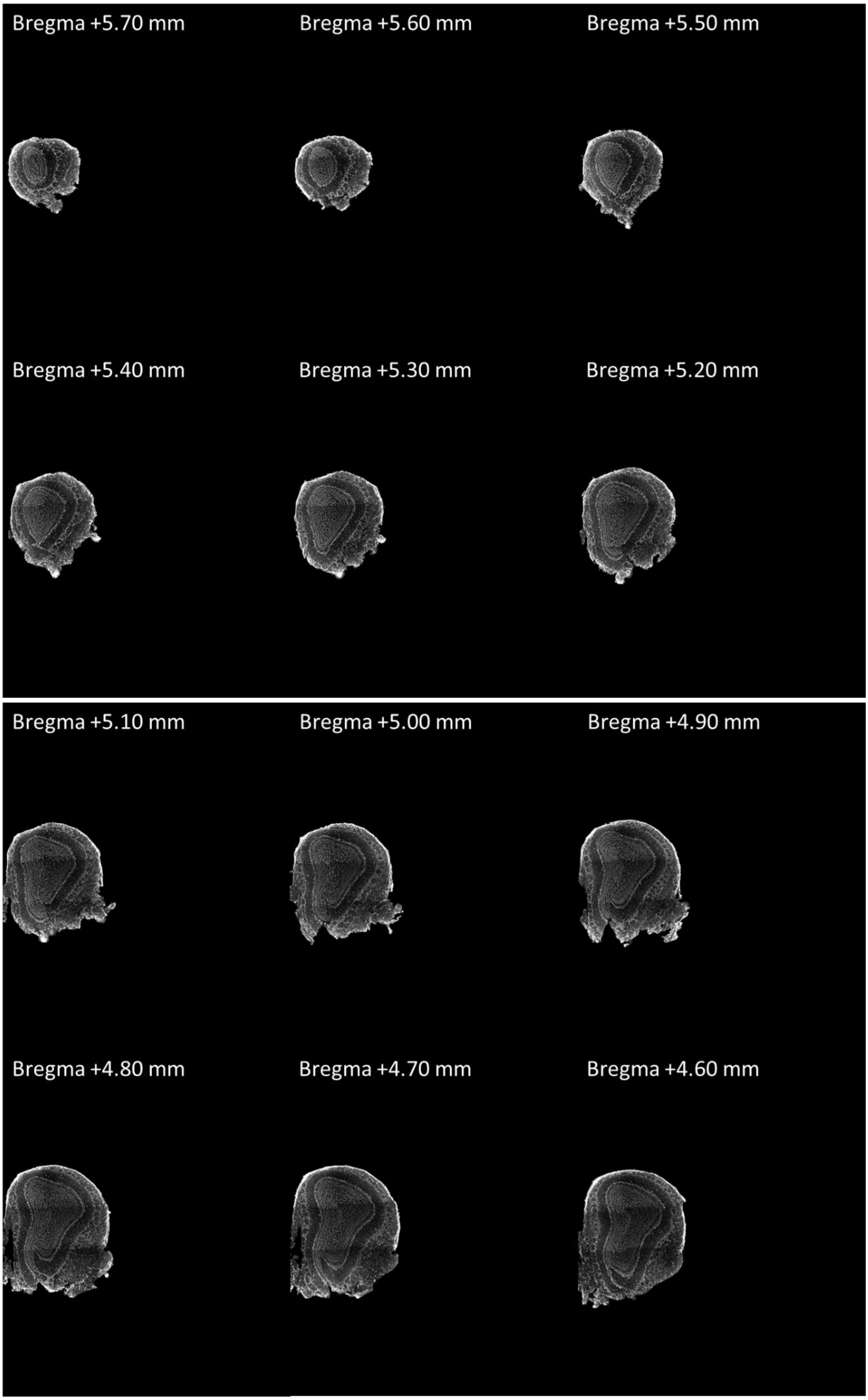
Bregma +5.70 mm
Bregma +5.60 mm
Bregma +5.50 mm
Bregma +5.40 mm
Bregma +5.30 mm
Bregma +5.20 mm
Bregma +5.10 mm
Bregma +5.00 mm
Bregma +4.90 mm
Bregma +4.80 mm
Bregma +4.70 mm
Bregma +4.60 mm

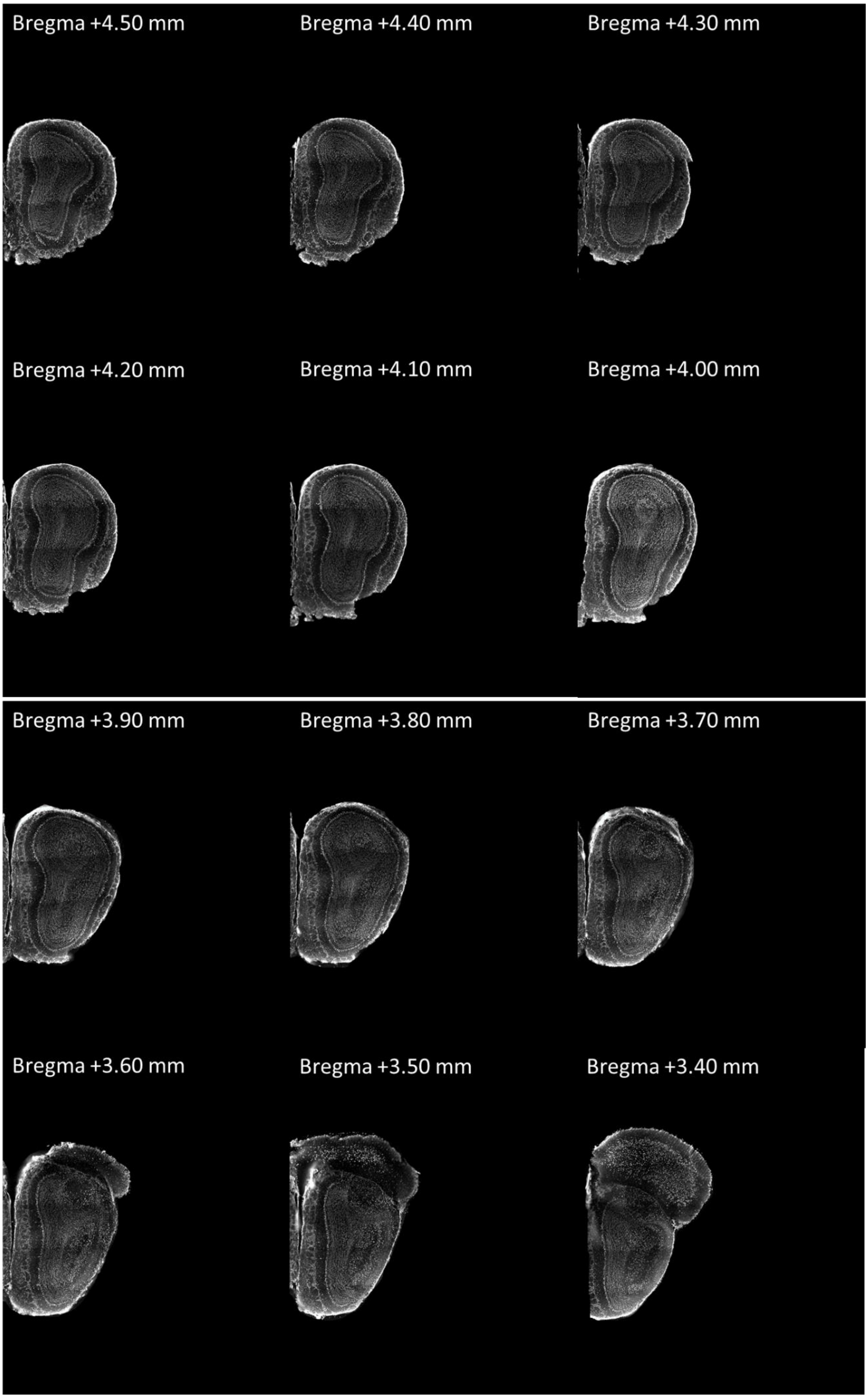
Bregma +4.50 mm
Bregma +4.40 mm
Bregma +4.30 mm
Bregma +4.20 mm
Bregma +4.10 mm
Bregma +4.00 mm
Bregma +3.90 mm
Bregma +3.80 mm
Bregma +3.70 mm
Bregma +3.60 mm
Bregma +3.50 mm
Bregma +3.40 mm

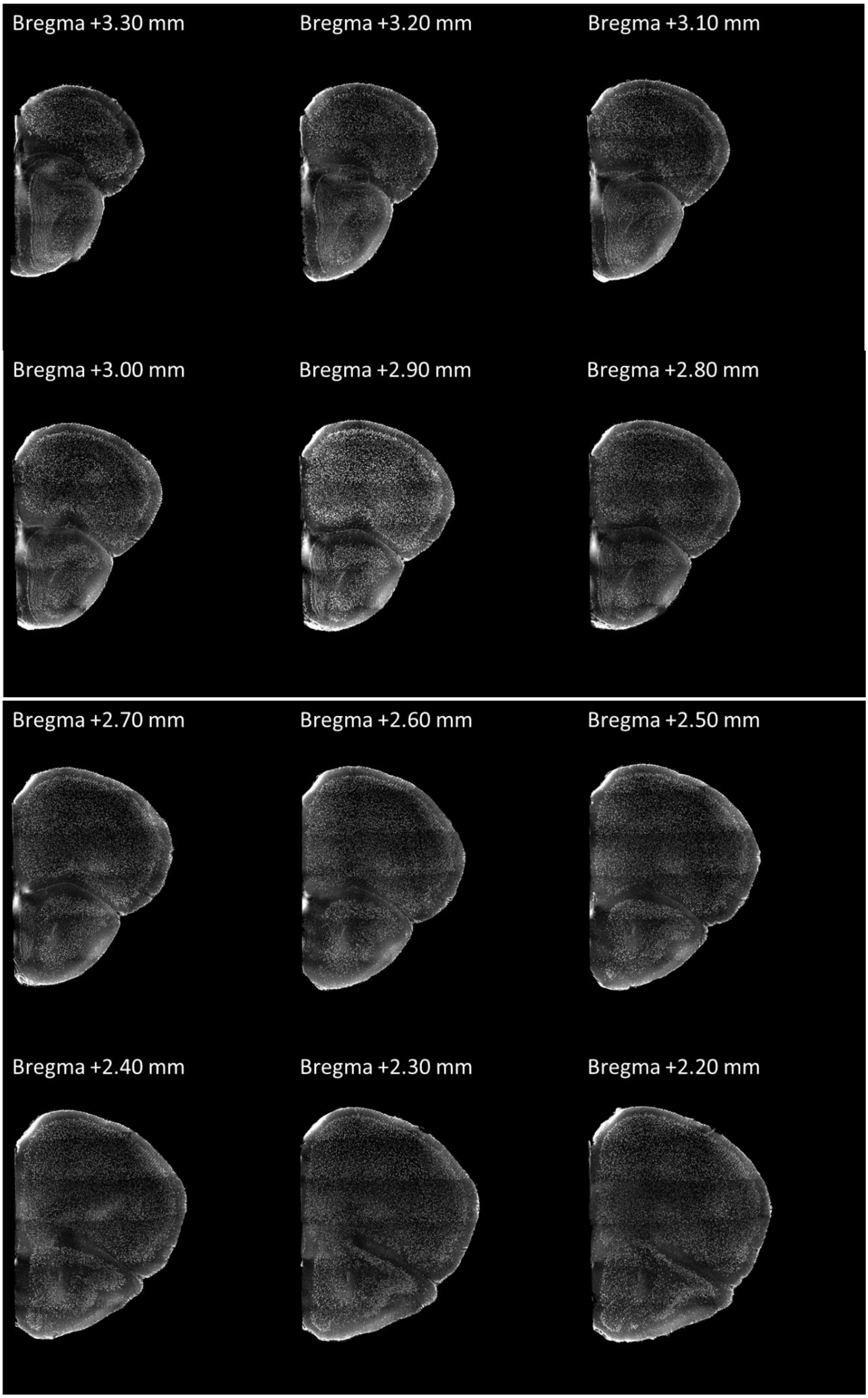
Bregma +3.30 mm
Bregma +3.20 mm
Bregma +3.10 mm
Bregma +3.00 mm
Bregma +2.90 mm
Bregma +2.80 mm
Bregma +2.70 mm
Bregma +2.60 mm
Bregma +2.50 mm
Bregma +2.40 mm
Bregma +2.30 mm
Bregma +2.20 mm

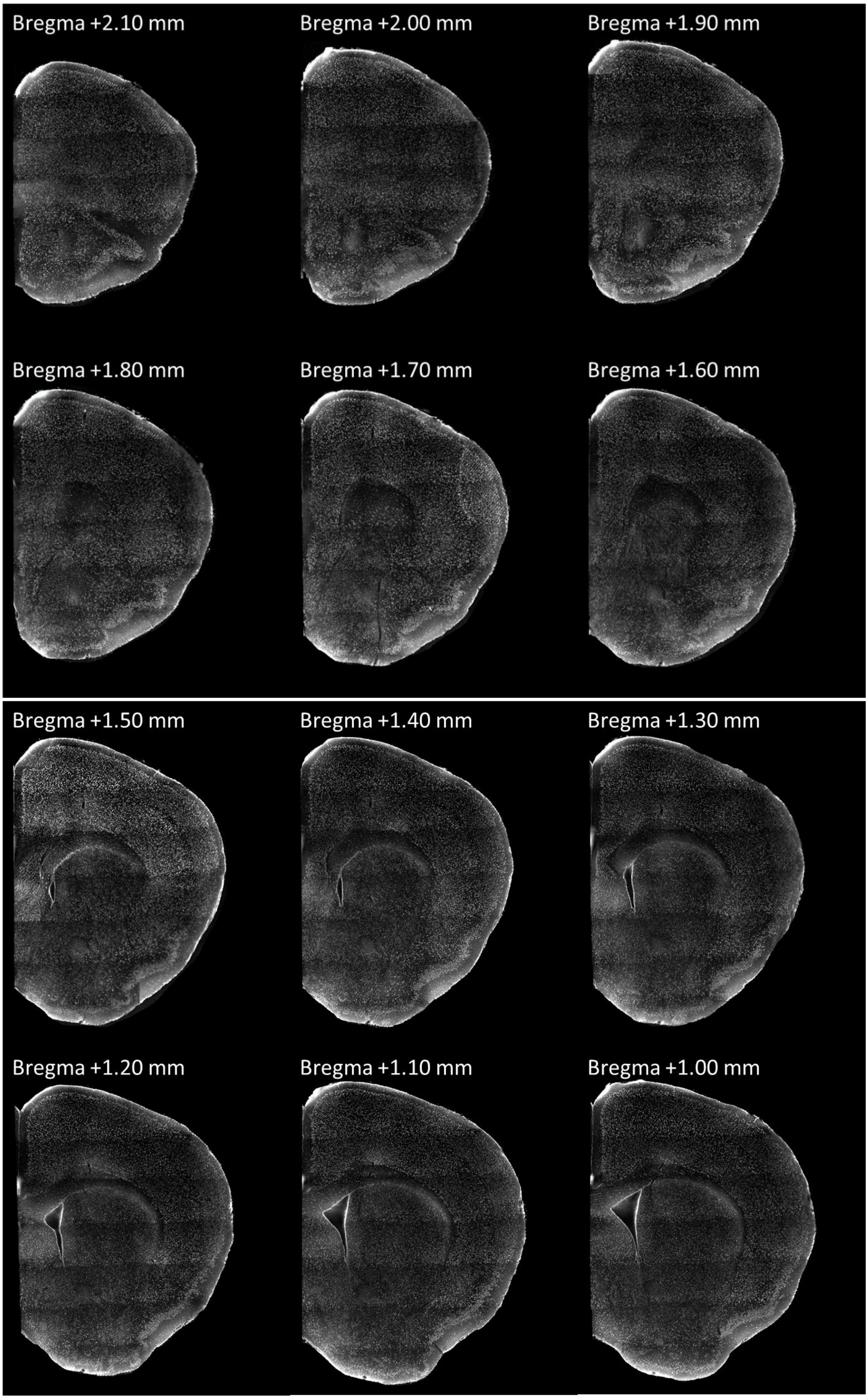
Bregma +2.10 mm
Bregma +2.00 mm
Bregma +1.90 mm
Bregma +1.80 mm
Bregma +1.70 mm
Bregma +1.60 mm
Bregma +1.50 mm
Bregma +1.40 mm
Bregma +1.30 mm
Bregma +1.20 mm
Bregma +1.10 mm
Bregma +1.00 mm

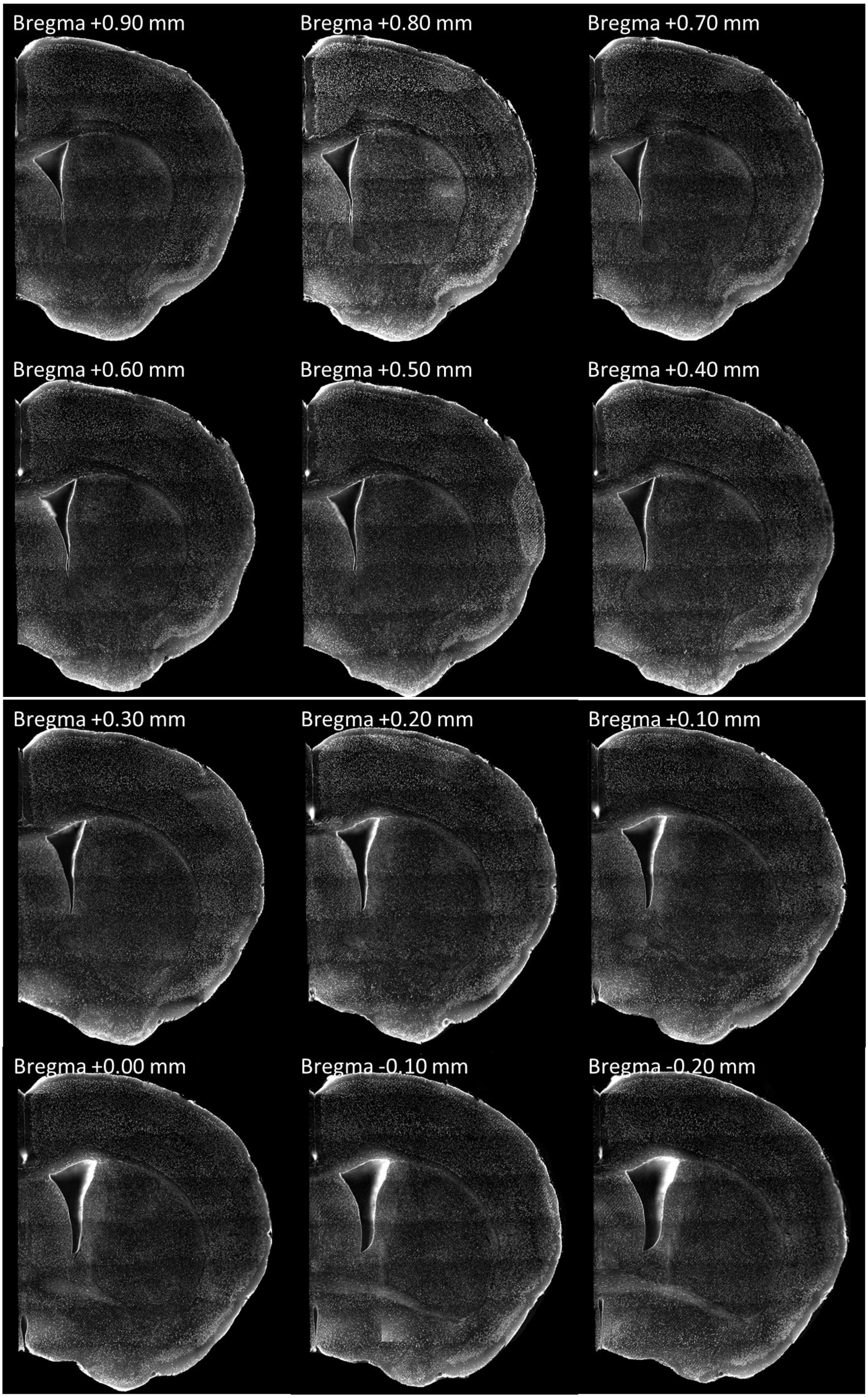
Bregma +0.90 mm
Bregma +0.80 mm
Bregma +0.70 mm
Bregma +0.60 mm
Bregma +0.50 mm
Bregma +0.40 mm
Bregma +0.30 mm
Bregma +0.20 mm
Bregma +0.10 mm
Bregma +0.00 mm
Bregma -0.10 mm
Bregma -0.20 mm

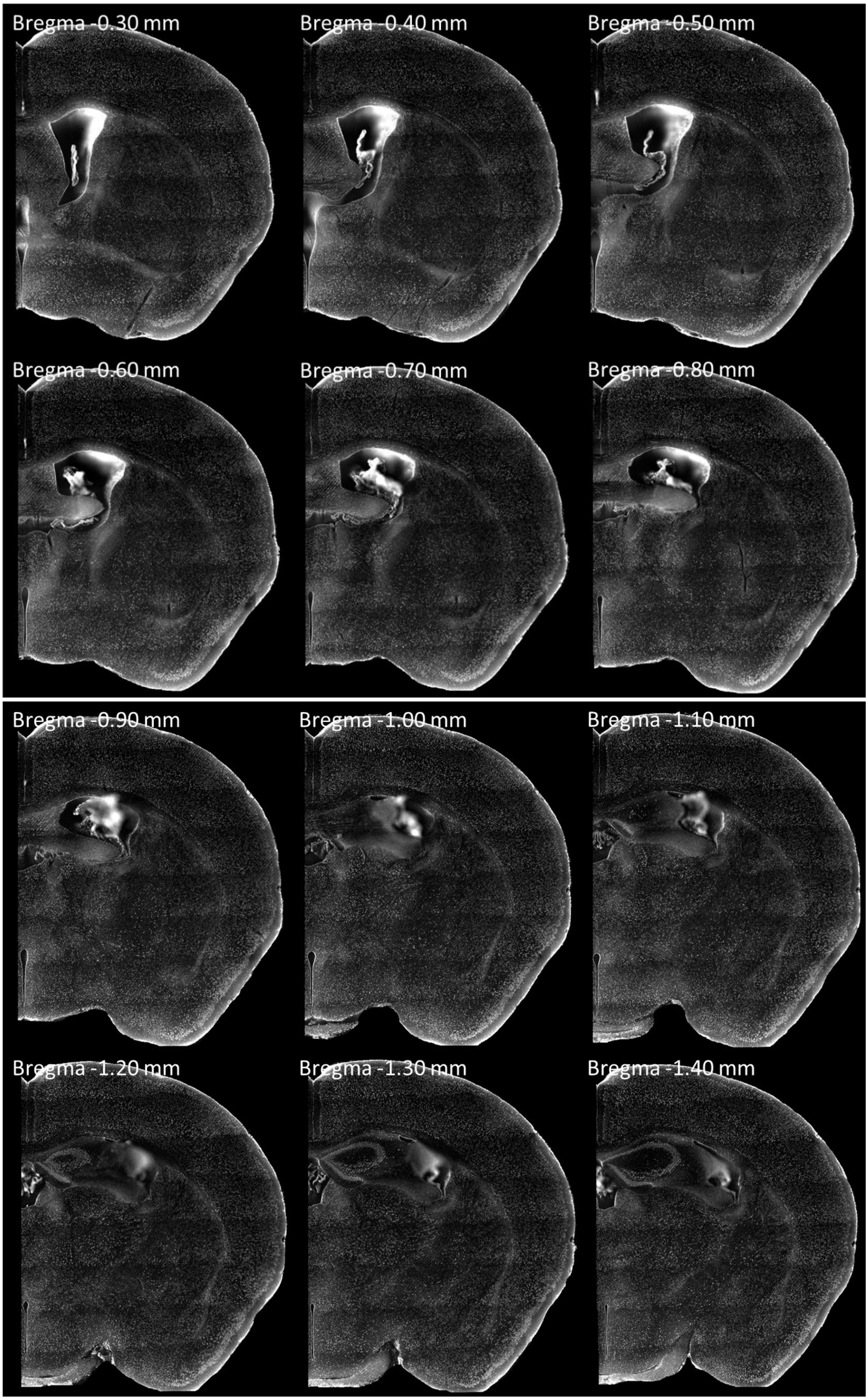
Bregma -0.30 mm
Bregma -0.40 mm
Bregma -0.50 mm
Bregma -0.60 mm
Bregma -0.70 mm
Bregma -0.80 mm
Bregma -0.90 mm
Bregma -1.00 mm
Bregma -1.10 mm
Bregma -1.20 mm
Bregma -1.30 mm
Bregma -1.40 mm

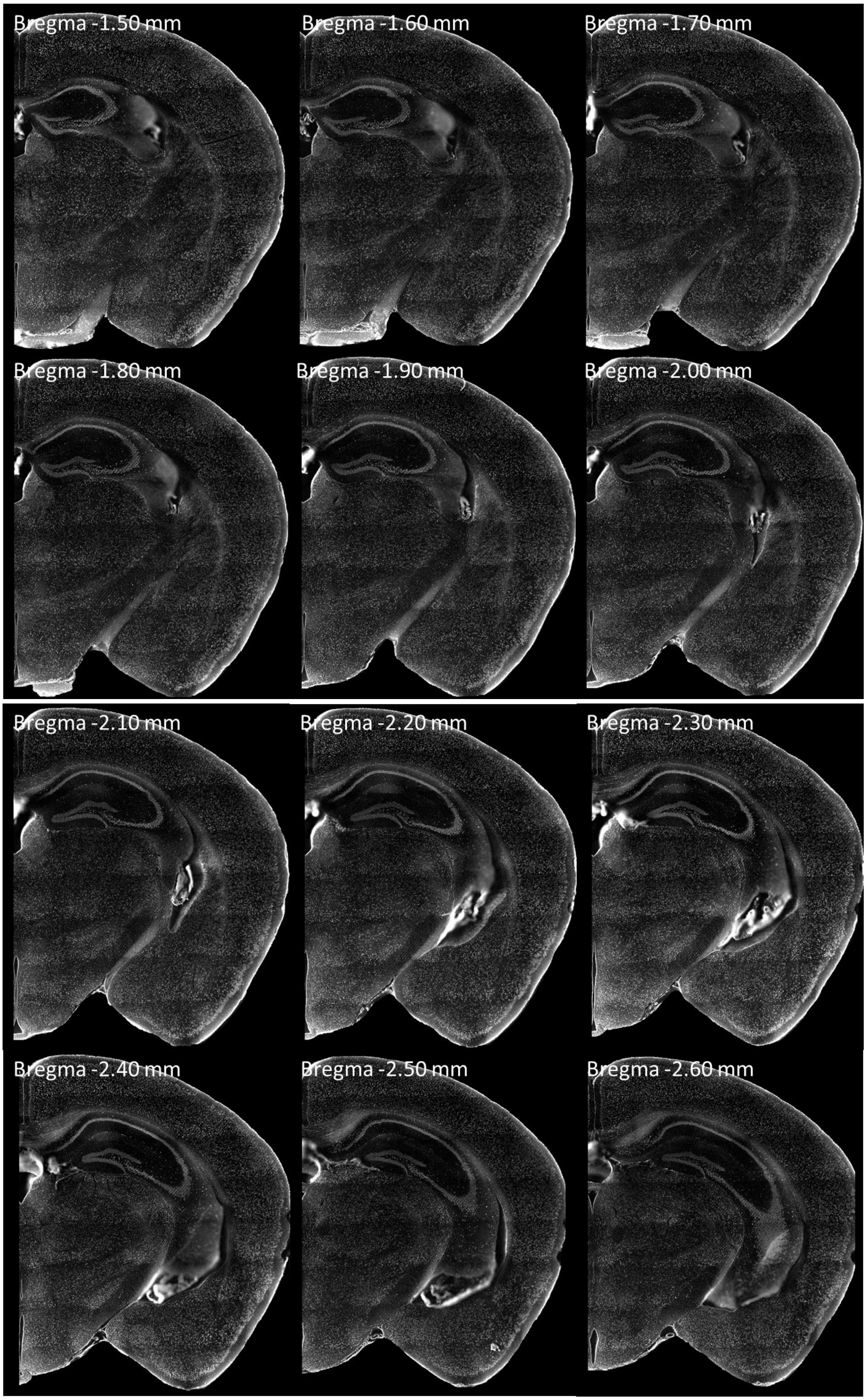
Bregma -1.50 mm
Bregma -1.60 mm
Bregma -1.70 mm
Bregma -1.80 mm
Bregma -1.90 mm
Bregma -2.00 mm
Bregma -2.10 mm
Bregma -2.20 mm
Bregma -2.30 mm
Bregma -2.40 mm
Bregma -2.50 mm
Bregma -2.60 mm

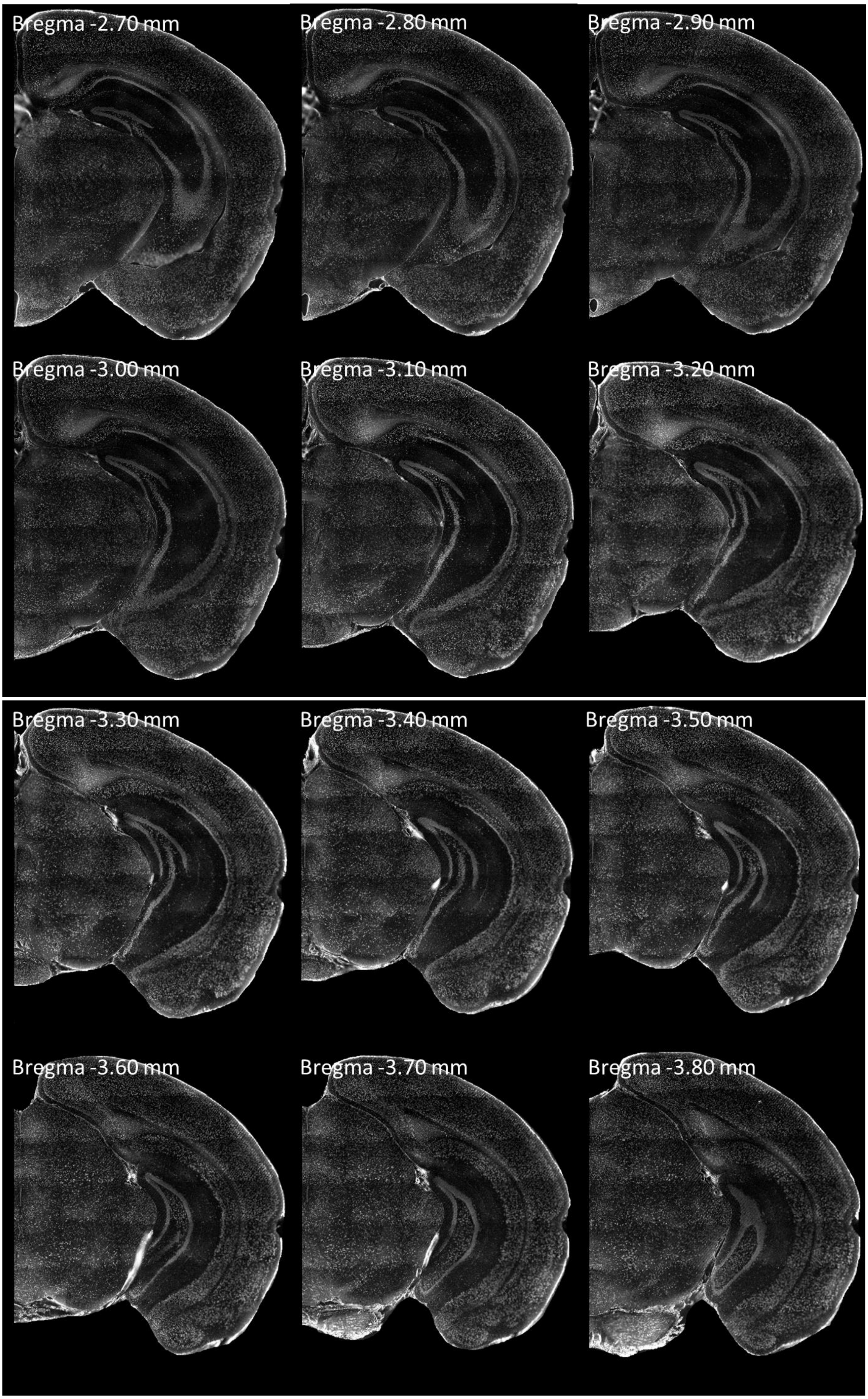

Bregma -2.70 mm
Bregma -2.80 mm
Bregma -2.90 mm
Bregma -3.00 mm
Bregma -3.10 mm
Bregma -3.20 mm
Bregma -3.30 mm
Bregma -3.40 mm
Bregma -3.50 mm
Bregma -3.60 mm
Bregma -3.70 mm
Bregma -3.80 mm

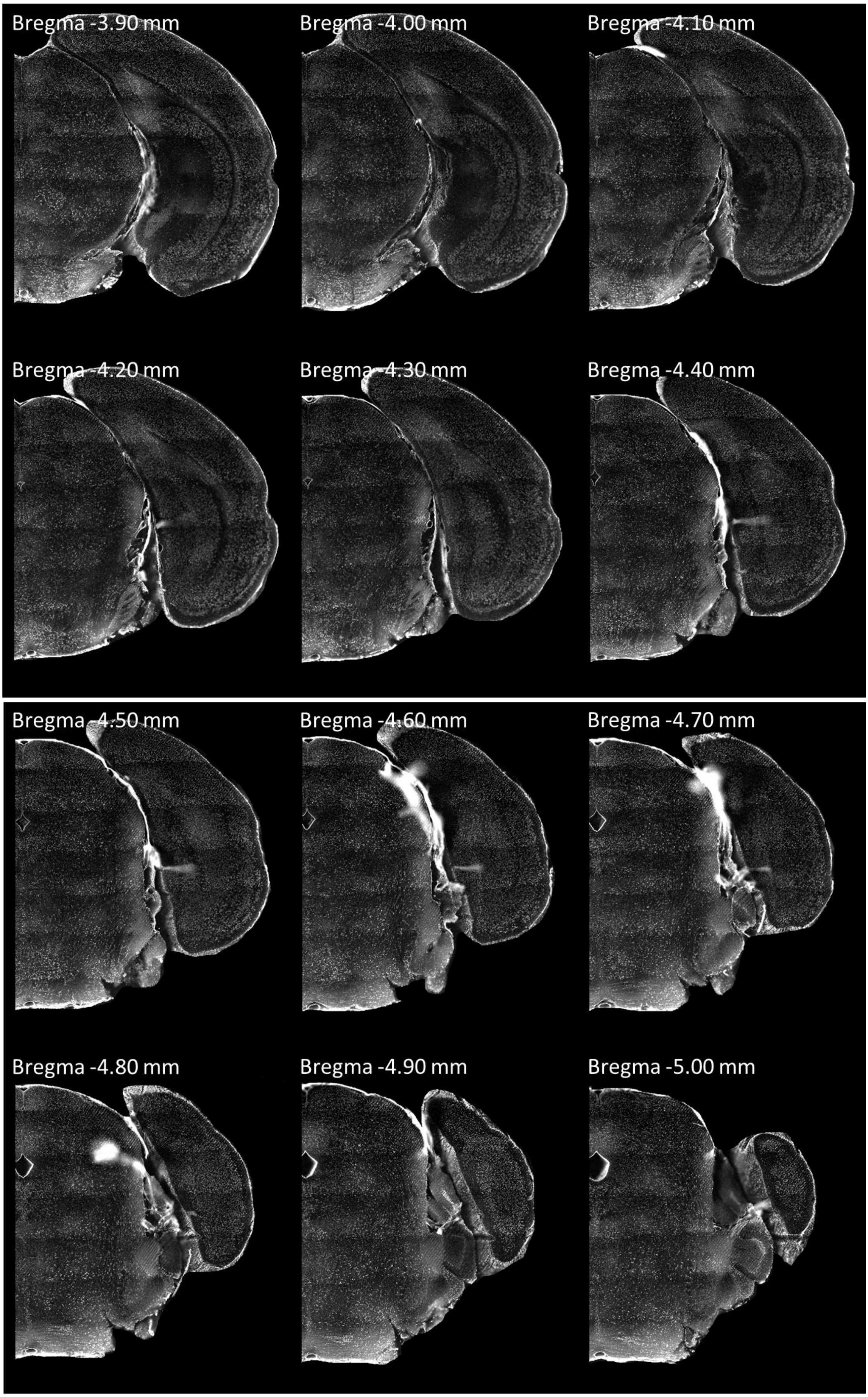
Bregma -3.90 mm
Bregma -4.00 mm
Bregma -4.10 mm
Bregma -4.20 mm
Bregma -4.30 mm
Bregma -4.40 mm
Bregma -4.50 mm
Bregma -4.60 mm
Bregma -4.70 mm
Bregma -4.80 mm
Bregma -4.90 mm
Bregma -5.00 mm

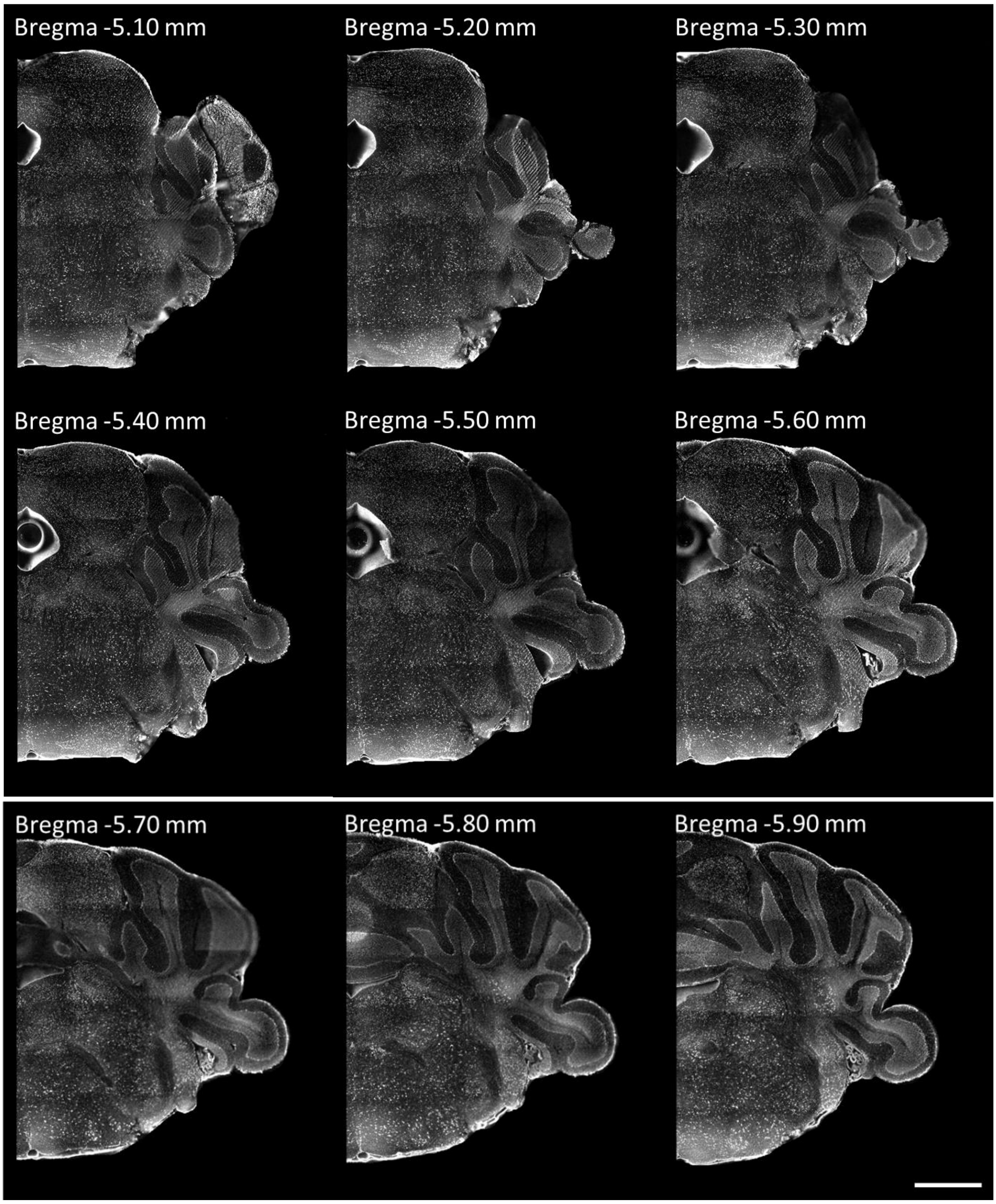

Supplementary Figure 2. High resolution images of the coronal sections shown in the montage in Figure 6. Scale bar, 1 mm.

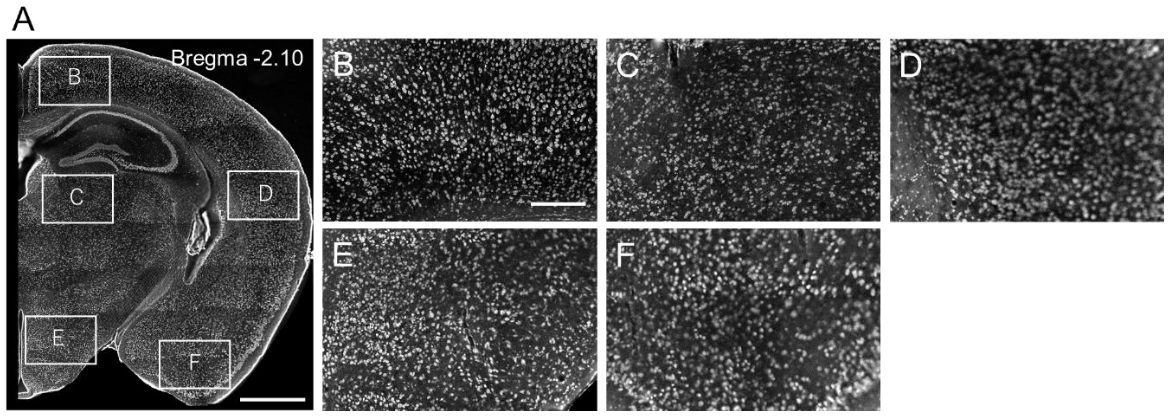

Supplementary Figure 3. A, A coronal section of the mouse brain stained with propidium iodide. B-F, Magnified views of the boxed area in panel A. Scale bars, 1 mm (A) and 200 µm (B, applies to panels C-F).

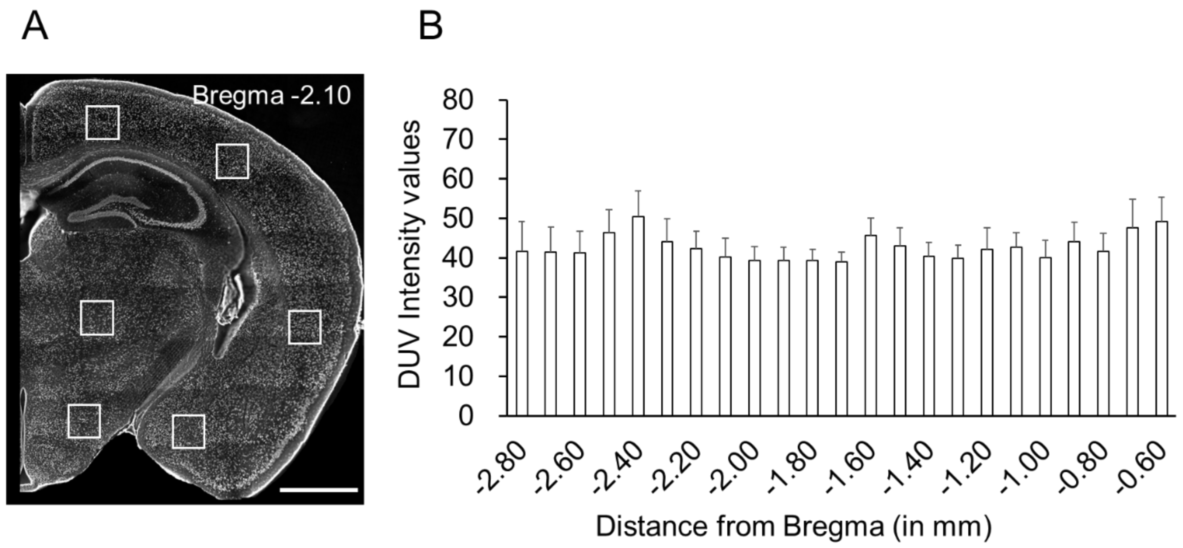

Supplementary Figure 4. A, A coronal section of the mouse brain stained with propidium iodide showing uniform staining irrespective of the brain regions. Boxes indicate rectangular field of views used to sample the signal intensity. B, A bar graph of the signal intensity in six rectangular field of views in each anterio-posterior level of the mouse brain. Values are presented as mean ± standard error of mean. Scale bar, 1 mm.
